# Angioedema as a Presenting Feature in a Patient With SLE: A Case Report

**DOI:** 10.7759/cureus.71939

**Published:** 2024-10-20

**Authors:** Tabinda Parvaiz Rasheed, Maryam Jabeen, Sheikh Ahsan Iqbal, Muhammad Khurram Saleem, Khawaja Faizan Ejaz, Amna Akbar, Sarosh Khan Jadoon, Mohammad Saleem Khan

**Affiliations:** 1 Internal Medicine, Abbas Institute of Medical Sciences (AIMS), Muzaffarabad, PAK; 2 Internal Medicine, Azad Jammu &amp; Kashmir Medical College, Muzaffarabad, PAK; 3 General Internal Medicine, University Hospitals Bristol and Weston NHS Foundation Trust, Bristol, GBR; 4 Internal Medicine, Russells Hall Hospital, Dudley, GBR; 5 Emergency and Accident, District Headquarters Hospital, Muzaffarabad, PAK; 6 General Surgery, Combined Military Hospital, Muzaffarabad, PAK; 7 Medicine, District Headquarters (DHQ) Teaching Hospital, Kotli, PAK

**Keywords:** acquired angioedema, angioedema, lupus, sle, systemic lupus erythematosus

## Abstract

Systemic lupus erythematosus (SLE) is a multi-organ autoimmune disease that can be easily missed due to its variable presentation. Acquired angioedema (AAE) is a rare first presentation of SLE. We report a case of a 23-year-old woman who presented to the emergency department with rapidly progressive swelling of the tongue and neck, followed by respiratory discomfort and a generalized non-itchy rash. A tracheostomy was performed to relieve her symptoms. Her history, examination, and relevant investigations all indicated SLE. She was treated with high-dose steroids, and pulse therapy with methylprednisolone was given for three days. Hydroxychloroquine was added, but she developed sepsis and an acute flare of SLE secondary to tracheostomy site infection. Subsequently, she was treated with broad-spectrum antibiotics, followed by a tapering dose of steroids and a maintenance dose of azathioprine. She responded to treatment, tracheostomy reversal was performed, and the patient was discharged.

## Introduction

Systemic lupus erythematosus (SLE) is an autoimmune disease in which the immune system of the body attacks its own tissues, resulting in widespread inflammation and tissue damage. It is a multi-organ disease that can affect the brain, skin, lungs, joints, blood vessels, and kidneys [1]. The mortality in SLE is 3.6 times higher than that in the general population due to its related complications [2]. Therefore, it is important to identify the various presentations of SLE to avoid negative outcomes.

Angioedema (AE), also called angioneurotic edema, is an acute-onset edema of the skin, subcutaneous tissue, and mucosa [3]. It differs from urticaria, in which skin eruptions are pruritic and can involve any area of the body, whereas AE preferentially involves the periorbital tissue and lips [4]. AE can be life-threatening if it causes swelling of the airway [5]. AE can be allergic (including anaphylaxis) or non-allergic. Non-allergic AE can be hereditary angioedema (HAE), acquired angioedema (AAE), pseudoallergic AE, drug-induced AE, or idiopathic AE [6]. HAE, usually associated with C1 esterase deficiency, has been documented in SLE patients before, but AAE is a rare complication of SLE and is an increasing concern [7,8].

We report the case of a 23-year-old woman with AAE as a presenting feature of SLE, highlighting the mistreatment by quacks that led to delays in diagnosis and timely aggressive management, worsening her prognosis.

## Case presentation

A 23-year-old woman was admitted to our emergency department with rapidly progressive swelling of the tongue and neck, followed by respiratory discomfort, inability to articulate, and a non-itchy rash predominantly involving dependent areas for one day. There was no history of any insect bite, food or drug allergies, or recent introduction of any new medications (angiotensin-converting enzyme (ACE) inhibitor, non-steroidal anti-inflammatory drugs (NSAIDs), aspirin, and antibiotics). The family history was not significant for any autoimmune disease or HAE. Upon systemic inquiry, she had a history of episodic swelling of the lips, oral mucosa, and oral ulcers, symmetrical polyarthritis involving small and large joints, photosensitivity, recurrent skin rash, and non-scarring alopecia over the past six years, for which she had been taking some unknown medications occasionally, as recommended by some quack/local Hakim.

**Figure 1:**
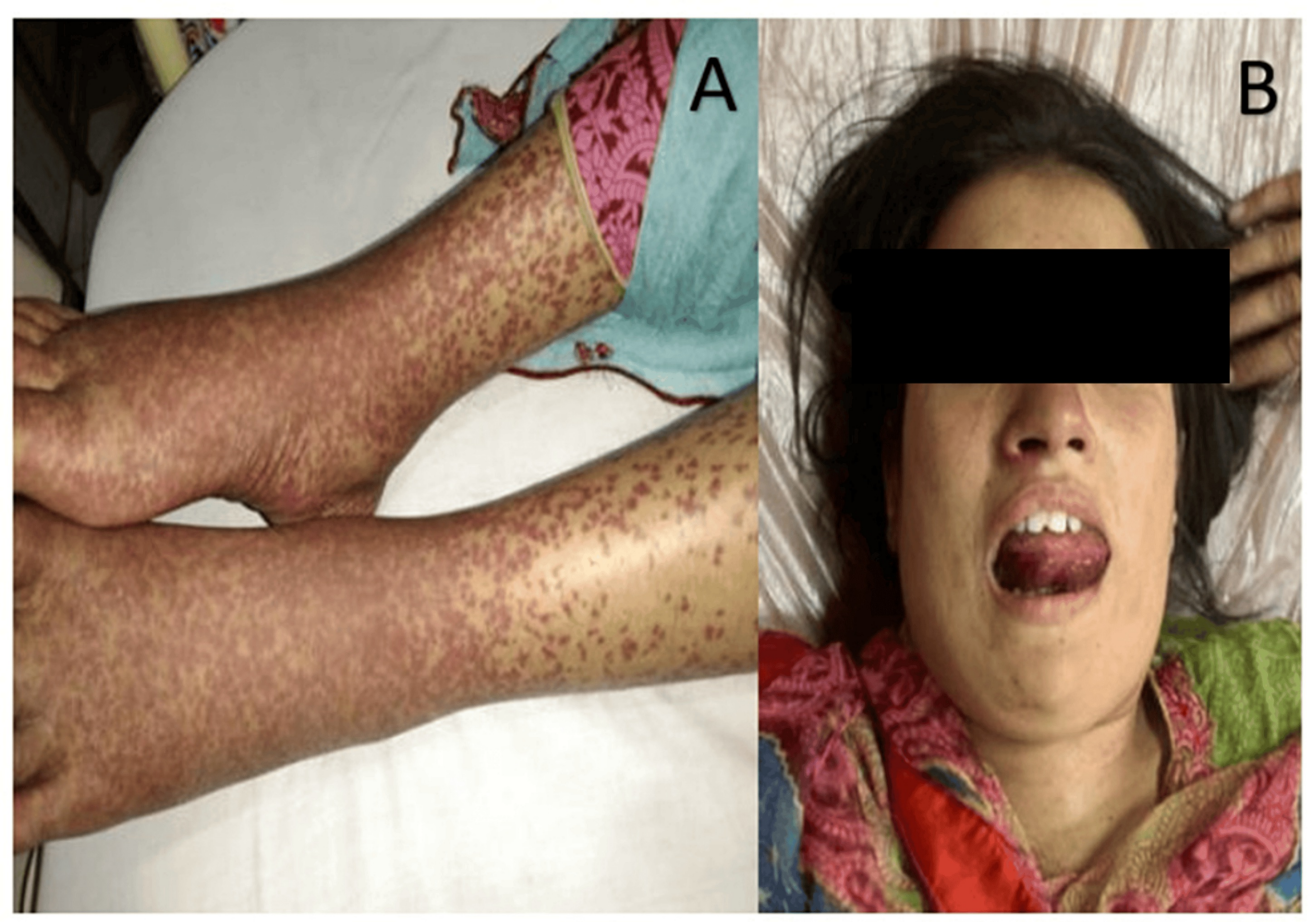
Examination findings in our patient. (A) Erythematous, non-blanchable, maculopapular rash involving the legs. (B) Edematous, erythematous, and protruding tongue with marked swelling of the face and neck.

At the time of admission, she was pale, restless, and tachypneic with obvious respiratory discomfort and a respiratory rate of 34 breaths per minute. She was tachycardiac, with a heart rate of 130 beats per minute, and her SpO_2_ was 92-94% on room air. She was afebrile and normotensive. Local examination revealed an edematous, erythematous, and protruding tongue with marked swelling of the face and neck. Cutaneous examination revealed an erythematous, palpable, non-blanchable, maculopapular rash predominantly involving the dependent areas, with no ulceration, necrosis, or nodules. Systemic examination was unremarkable, as shown in Figure 1.

Laboratory investigations showed bicytopenia with a hemoglobin (Hb) level of 9.9 g/dL, platelet count of 145 x 10^9^/L, and increased leukocyte count (TLC). Liver function tests (LFTs) showed increased total serum bilirubin (TSB), alanine transaminase (ALT), and alkaline phosphatase (ALP) levels. Renal function tests (RFTs) indicated increased urea and creatinine levels. Serum sodium (Na+) and chloride (Cl-) levels were within the normal limits, but serum potassium (K+) levels were mildly elevated. The prothrombin time/activated partial thromboplastin time (PT/APTT) was within normal limits. Hepatitis serology tests for B and C were negative. Electrocardiography (ECG) revealed sinus tachycardia. Anti-double-stranded DNA (DsDNA) antibodies and anti-Ro/anti-LA antibodies were positive. Complement levels C3 and C4 were low, erythrocyte sedimentation rate (ESR) was 60 mm in the first hour, and 24-hour urine test showed 2.3 g of protein per 24 hours. The remaining investigations were within normal limits, as summarized in Tables 1, 2.

**Table 1:** Laboratory investigations. CBC: complete blood count; Hb: hemoglobin; TLC: total leukocyte count; Plt: platelet count; LFTs: liver function tests; TSB: total serum bilirubin; ALT: alanine transaminase; ALP: alkaline phosphatase; RFTs: renal function tests; PT: prothrombin time; APTT: activated partial thromboplastin time.

Parameter	Result	Reference value
CBC		
Hb	9.9 g/dL	11–14.5
TLC	13 x 10^9^/L	4.6–10.8
Plt	145 x 10^9^/L	154–433
LFTs		
TSB	1.1 mg/dL	0.1 – 1.2
ALT	97 IU/L	0 – 55
ALP	572 IU/L	45 – 129
RFTs		
Urea	77 mg/dL	10–50
Creatinine	2.5 mg/dL	0.6–1.1
Serum electrolytes		
Sodium (Na+)	136 mmol/L	136–145
Chloride (Cl-)	106 mmol/L	98–107
Potassium (K+)	5.6 mmol/L	3.5–5.1
PT	11.0 seconds	9.3–12.8
APTT	33.0 seconds	22.9–34.5
Bicarbonate (HCO3-)	22.8 mmol/L	20–31
Serum glucose	124 mg/dL	80–160

**Table 2:** Laboratory investigations. ANCA: antineutrophil cytoplasmic antibodies; RNP: ribonucleoprotein.

Parameter	Result
Anti-DsDNA antibodies	Positive
Anti-Ro/anti-LA antibodies	Positive
Anti-RNP antibodies	Negative
C3 and C4 levels	Low
c-ANCA, p-ANCA	Negative
Coombs test (direct and indirect)	Negative
2D echo	Normal
Blood and urine cultures	No yield

Based on history, examination, and laboratory data, the Systemic Lupus International Collaborating Clinics (SLICC) criteria were used [9], and a diagnosis of AAE secondary to SLE was established (Table 3).

**Table 3:** SLICC criteria. The presence of four features with equal or more than one clinical feature and one immunological feature OR biopsy-proven lupus nephritis with anti-DsDNA antibodies or ANA. ANA: antinuclear antibody; APA: antiphospholipid antibody; DsDNA: double-stranded DNA; Sm: Smith; SLICC: Systemic Lupus International Collaborating Clinic.

Clinical features		Immunological features	
1.	Acute cutaneous lupus (maculopapular lupus rash, malar rash, photosensitive lupus rash, etc.)	1.	High ANA concentration
2.	Chronic cutaneous lupus (discoid rash, mucosal lupus, etc.)	2.	High anti-DsDNA antibody concentration
3.	Oral or nasal ulcers	3.	Presence of anti-Sm
4.	Non-scarring alopecia	4.	Positive APA
5.	Synovitis in two or more joints	5.	Low complement (C3, C4, CH50)
6.	Serositis	6.	Direct Coombs test
7.	Renal (urine proteins or RBC casts)		
8.	Neurologic (seizures, psychosis, others)		
9.	Hemolytic anemia		
10.	Leukopenia or lymphopenia (without an identifiable cause)		
11.	Thrombocytopenia (without an identifiable cause)		

With the help of ENT department, an immediate urgent tracheostomy was performed to secure the airway, and the patient was shifted to the intensive care unit (ICU). On the day of admission, she was administered broad-spectrum intravenous (IV) antibiotics, IV antihistamines, IV steroids (hydrocortisone), and IV sedatives. Methylprednisolone (1 g/day) was initiated on the second day of admission and continued for three days. Oral steroids (prednisolone, 1 mg/kg/day) and hydroxychloroquine (4 mg/kg/day) were added on the fourth day of admission. A nephrologist consultation was also taken on board regarding proteinuria and deranged renal functions; he agreed with already advised treatment plan. A C1 esterase inhibitor (C1-INH) was planned but could not be given due to its non-availability. After stabilizing the patient, steroids were tapered down, but she developed an acute flare of SLE with worsening rash, systemic toxicity, and sepsis on the ninth day of admission due to an abscess at the tracheostomy site and herpes labialis. Careful pus drainage and wound care were provided by the ENT department. Another course of broad-spectrum antibiotics was administered after modifying according to culture sensitivity along with oral antivirals. Subsequently, maintenance steroids were administered and gradually titrated, and azathioprine (50 mg/day) was initiated one day before discharge. She was discharged on low-dose oral corticosteroids for four weeks, followed by maintenance therapy with azathioprine. There was a marked improvement in her condition, and the AE resolved. Therefore, tracheostomy reversal was performed. Hemoglobin level improved to normal, proteinuria resolved, and sepsis resolved. Currently, her disease is under control, and the patient is kept under close follow-up.

## Discussion

SLE is an autoimmune disease with a reported prevalence of 20-150 cases per 100,000 individuals in the United States. In South America, North America, Asia, and Europe, the estimated incidence rates are 1-25 per 100,000 individuals [10]. It is more common in young females, especially those of childbearing age [11]. SLE can have variable presentations in clinical settings, including constitutional symptoms (fever, fatigue, weight change), renal involvement (lupus nephritis), cardiac involvement (myocardial infarction, pericarditis, thrombosis, cardiac tamponade), pulmonary involvement (pulmonary edema/hemorrhage, pulmonary hypertension), neurological involvement (psychosis, stroke, seizures), gastrointestinal involvement (esophagitis, hepatitis, acute pancreatitis), mucocutaneous involvement (malar rash, discoid lesion), and arthritis [11,12]. Due to these diverse variations in presentations, SLE can be missed or misdiagnosed and easily mistreated. Fatigue, fever, arthralgia, and weight changes are the most common symptoms of presentation. According to one meta-analysis, it was found that articular manifestations, pulmonary involvement, and pleuritis were significantly more common in adults than in children [13]. Approximately 50% of patients with SLE develop clinically evident kidney disease.

AE, also called angioneurotic edema, is an acute-onset edema of the skin, subcutaneous tissue, and mucosa that commonly affects the periorbital region and lips [3,4]. It can be allergic/mast cell-mediated or non-allergic/kinin-mediated. Kinin-mediated AE occurs due to vasodilation and increased permeability caused by mediators of inflammation, especially bradykinin, whose production is regulated by C1-INH. C1-INH is an inhibitor of the complement pathway and plasma kallikrein-kinin system, which is responsible for the release of bradykinin [14]. According to a case-based review, 18 cases of AAE associated with lupus have been reported so far [15]. Kinin-mediated AE can be divided into hereditary and acquired forms. There are two types of HAE, depending on C1-INH mutations that can affect either its secretion or function [16]. There is a third type in which C1-INH levels and functions are normal, and it occurs only in females [17,18].

AAE was first reported in 1972 [19]. AAE is associated with lymphoproliferative disorders, infections, neoplasms, and autoimmune diseases such as SLE [20]. AAE is less frequent than HAE. A study indicated that HAE can be present in approximately 2% of lupus cases, whereas AAE can be present in less than 1% of cases [21]. AAE can occur in two types. In type 1 AAE, there is catabolism of C1INH due to lymphoproliferative disorders, leading to decreased levels of C1-INH. In type 2, there is an autoantibody against C1-INH, whereas levels of inactive C1-INH can be normal or elevated [20,22]. A third type of AAE has been described with transiently low levels of C1-INH, hypocomplementemia (low levels of C3 and C4), and the absence of antibodies against C1-INH [23]. Patients with SLE may have type 2 or type 3 AAE.

Our patient had AAE secondary to SLE. A previous history of photosensitivity, polyarthritis, episodic swelling of the lips and oral mucosa, oral ulcers, and non-scarring alopecia all can indicate SLE. Suppression of these symptoms was done with some unknown medications prescribed by the quacks/local Hakim. Although the patient did not have medications with her, we assumed them to be steroids because of their positive response in her case and the common malpractice by quacks in our localities. Due to the initial improvement, the patient did not consult a healthcare setup for her symptoms and continued them until she presented with acute flare and severe AE. The absence of a positive family history and advanced age ruled out HAE. SLE was diagnosed using the SLICC criteria. There were positive anti-DsDNA antibodies and anti-Ro/anti-LA antibodies, and low levels of C3 and C4 were detected. The type of AAE was not identified in our patient due to the non-availability of investigations for C1-INH and antibodies in our setup. Since the patient had laryngeal spasm, urgent tracheostomy was performed, indicating the need for airway maintenance irrespective of the cause. During the hospital stay, the patient developed an acute flare of SLE due to infection of the tracheostomy site and herpes labialis. Studies have shown that infection is one of the causes of lupus flare [24,25]. With timely and appropriate treatment, the patient improved and was discharged with strict follow-up.

This case report also highlights the importance of awareness of autoimmune diseases and their symptoms, especially in young females, so that they report to the healthcare setup for proper investigations and treatment instead of going to quacks and local malpractitioners. AE in SLE is a serious but treatable condition if reported in time. With proper management of SLE and education on the disease, the prognosis can be improved in patients, and complications can be prevented.

## Conclusions

SLE should be considered in the differential diagnosis, especially in young females of childbearing age presenting with new-onset AE. Timely diagnosis and aggressive management of such cases can reduce patient mortality and morbidity.
